# Counseling and Educating About Vasa Previa Using Intraoperative Photographs of Vessel Rupture: A Case Report

**DOI:** 10.7759/cureus.79964

**Published:** 2025-03-03

**Authors:** Allan Barraza, Amy J Gagnon

**Affiliations:** 1 Obstetrics and Gynecology, Intermountain Health - Saint Joseph Hospital, Denver, USA; 2 Maternal Fetal Medicine, Kaiser Permanente, Denver, USA

**Keywords:** fetal exsanguination, high-risk obstetrics, interprofessional education and collaboration, medical education, nursing education and practice, vasa previa

## Abstract

Vasa previa describes vessels that are unprotected by Wharton’s jelly and traverse over or near the endocervix. Rupture of membranes or labor increases the risk of vessel rupture, followed by rapid fetal exsanguination. Learners are often taught to manage this condition by case scenarios and simulations. We present a case of type I vasa previa where intraoperative photographs demonstrate the potentially catastrophic rupture of membranes into the unprotected fetal vessels during a scheduled cesarean delivery. We recommend using intraoperative photographs to educate learners and counsel patients on the potential acuity inherent to this condition.

## Introduction

Vasa previa describes fetal vessels that are not covered by Wharton’s jelly and traverse over or proximal to the endocervix [1]. Vasa previa affects around 1 in 2,500 births and is classified into three types [1]. Type I vasa previa occurs when a velamentous placental cord insertion has unprotected fetal vessels within the membranes over or near the endocervix [1]. Type II vasa previa occurs when unprotected fetal vessels connecting a succenturiate or multilobed placenta traverse over or near the endocervix [1]. Type III vasa previa occurs when unprotected vessels connecting two placental edges traverse over or near the endocervix [2]. Vasa previa incurs the risk of fetal exsanguination at the time of membrane rupture or onset of labor. For this reason, inpatient surveillance and antenatal corticosteroids are considered as early as 28 weeks, followed by planned cesarean delivery between 34 and 37 weeks [1,3].

Neonatal survival for vasa previa is as high as 97.6%, largely secondary to improvements in prenatal diagnosis at the second-trimester ultrasound [1]. Mortality approaches 56% when vasa previa is not diagnosed antenatally [1]. Careful surgical skill during cesarean delivery is also essential for a good outcome, with surgeons being mindful of the location of the unprotected fetal vessels. This can be accomplished by exposing the membranes and incising them away from the vessels or attempting fetal delivery en caul [2]. Perioperative ultrasound mapping may also be considered [2,4].

However, prolonged antepartum admissions are known to cause social and financial hardship on patients, many of whom also have jobs and young children [5,6]. One in three antepartum patients have clinically significant anxiety and depression, twice the prevalence of the general obstetric population [4]. Asymptomatic patients with vasa previa may be hesitant to the recommendation for admission due to these socioeconomic implications. Thorough counseling by providers is imperative to achieving patient engagement and shared decision making. 

Providers, caregivers, and support staff are educated on vasa previa by written case scenarios and simulation exercises. While these activities help care teams understand the management of vasa previa, there is a potential gap in illustrating the acuity inherent to this diagnosis. We present a case where intraoperative photographs of vessel rupture during a scheduled cesarean section were used for patient counseling and multidisciplinary education on the acuity of a ruptured vasa previa. 

## Case presentation

**Figure 1:**
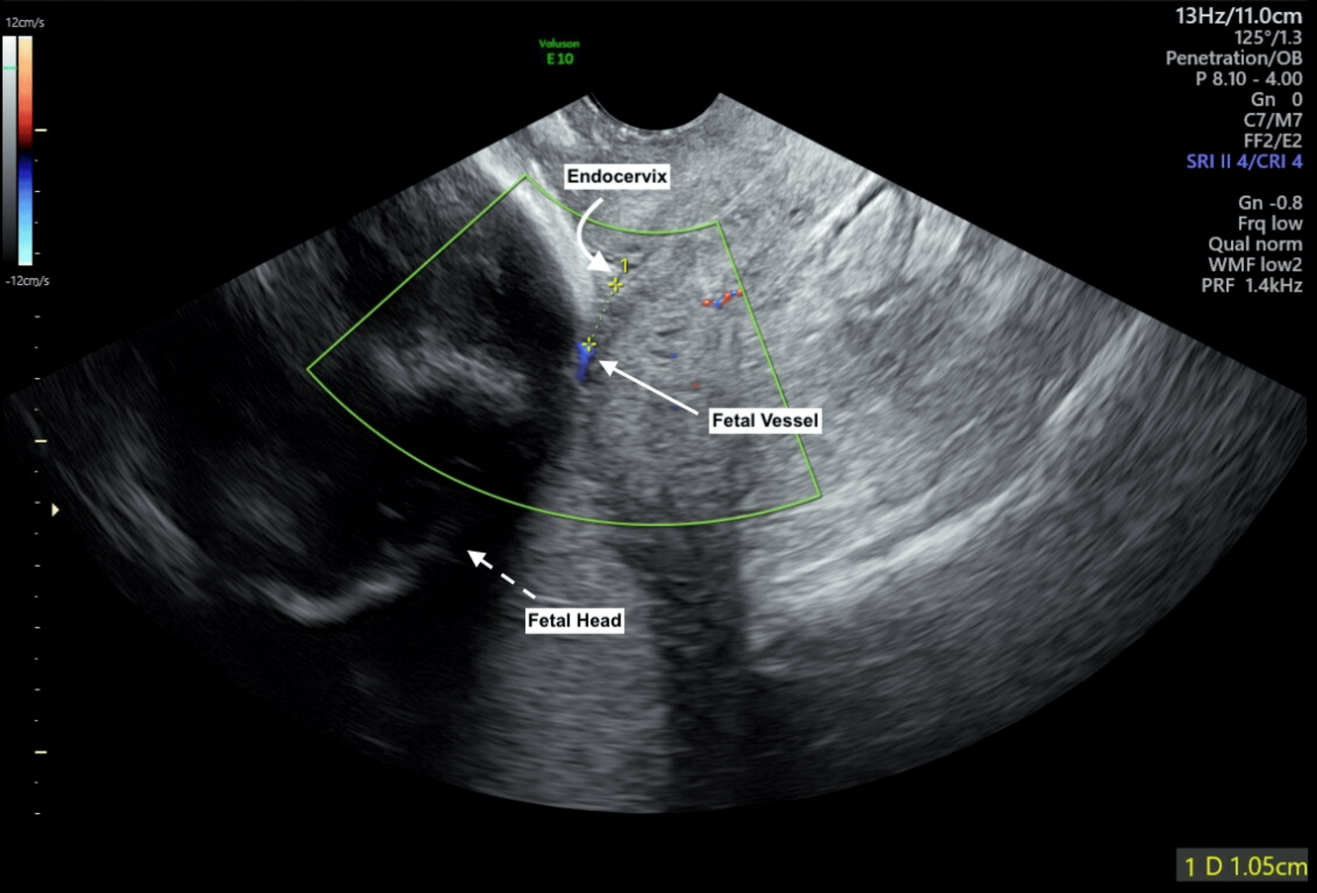
Transvaginal ultrasound using color Doppler to locate unprotected fetal vessels in type 1 vasa previa. Transvaginal ultrasound at 32 weeks gestation where color Doppler identified an unprotected fetal vessel (straight arrow) overlying the fetal head (dotted arrow) at 1.05 cm from the endocervix (curved arrow).

A 30-year-old gravida 2 para 1001 at 22 weeks gestation was referred to Maternal Fetal Medicine (MFM) following a second trimester screening ultrasound where fetal vessels were overlying the endocervix. An MFM ultrasound confirmed the diagnosis of type I vasa previa, with a velamentous cord insertion at 1.9 cm from the endocervix. Serial interval ultrasounds confirmed persistent type I vasa previa with these vessels lying at 1.05 cm from the endocervix at 32 weeks gestation (Figure 1).

The patient was admitted at 32 weeks gestation for inpatient surveillance. At admission, she received a course of antenatal corticosteroids and fetal monitoring three times per day. A shared decision was made by the obstetrician, MFM, and the patient to schedule a cesarean delivery at 35 weeks with intraoperative photographs to document the vasa previa. 

**Figure 2:**
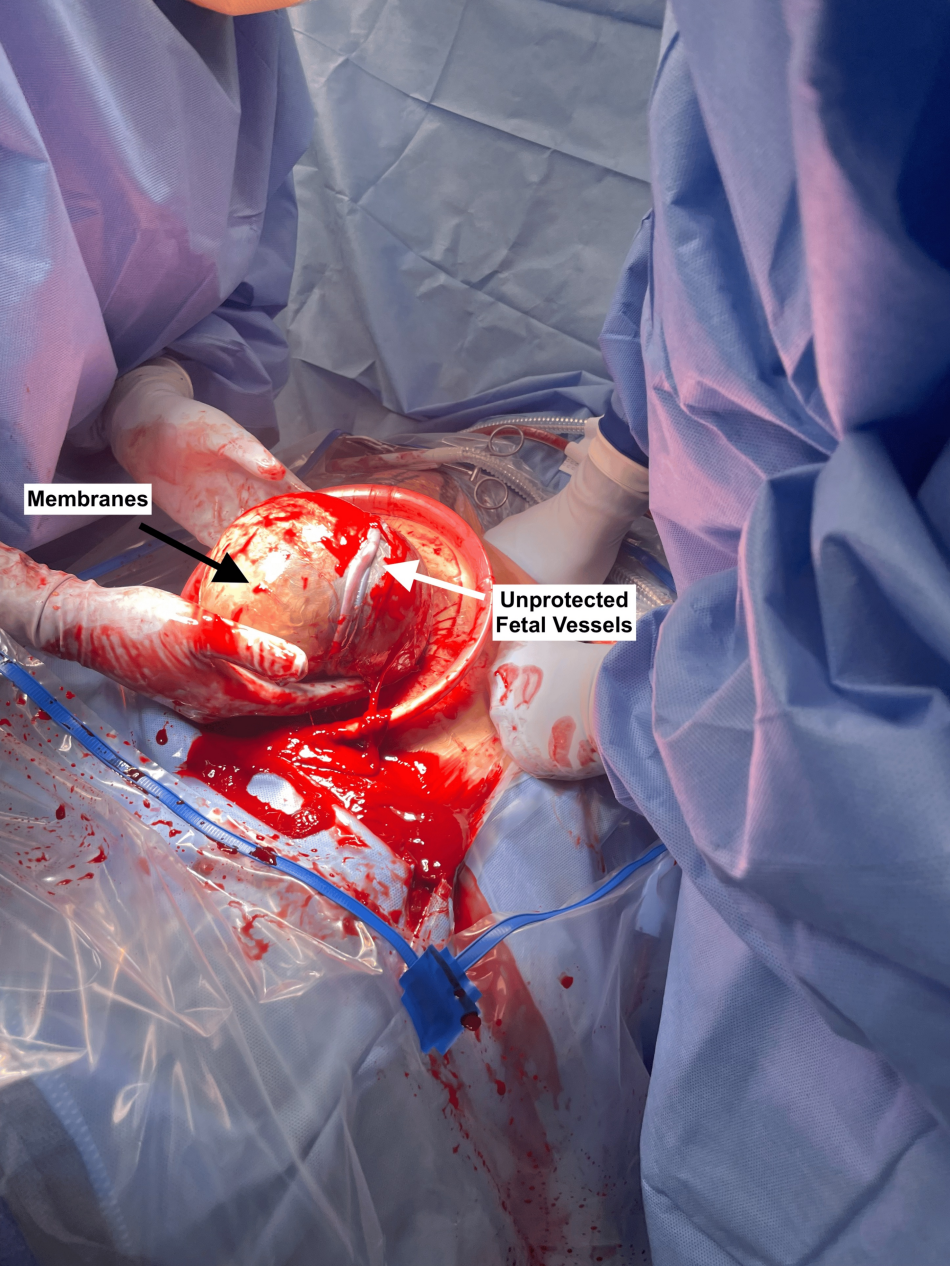
Attempted en caul delivery for type I vasa previa. Surgeons attempting an en caul cesarean delivery, where the fetus is delivered with unruptured membranes. The unprotected fetal vessels (white arrow) are overlying the fetal head, surrounded by intact membranes (black arrow).

**Figure 3:**
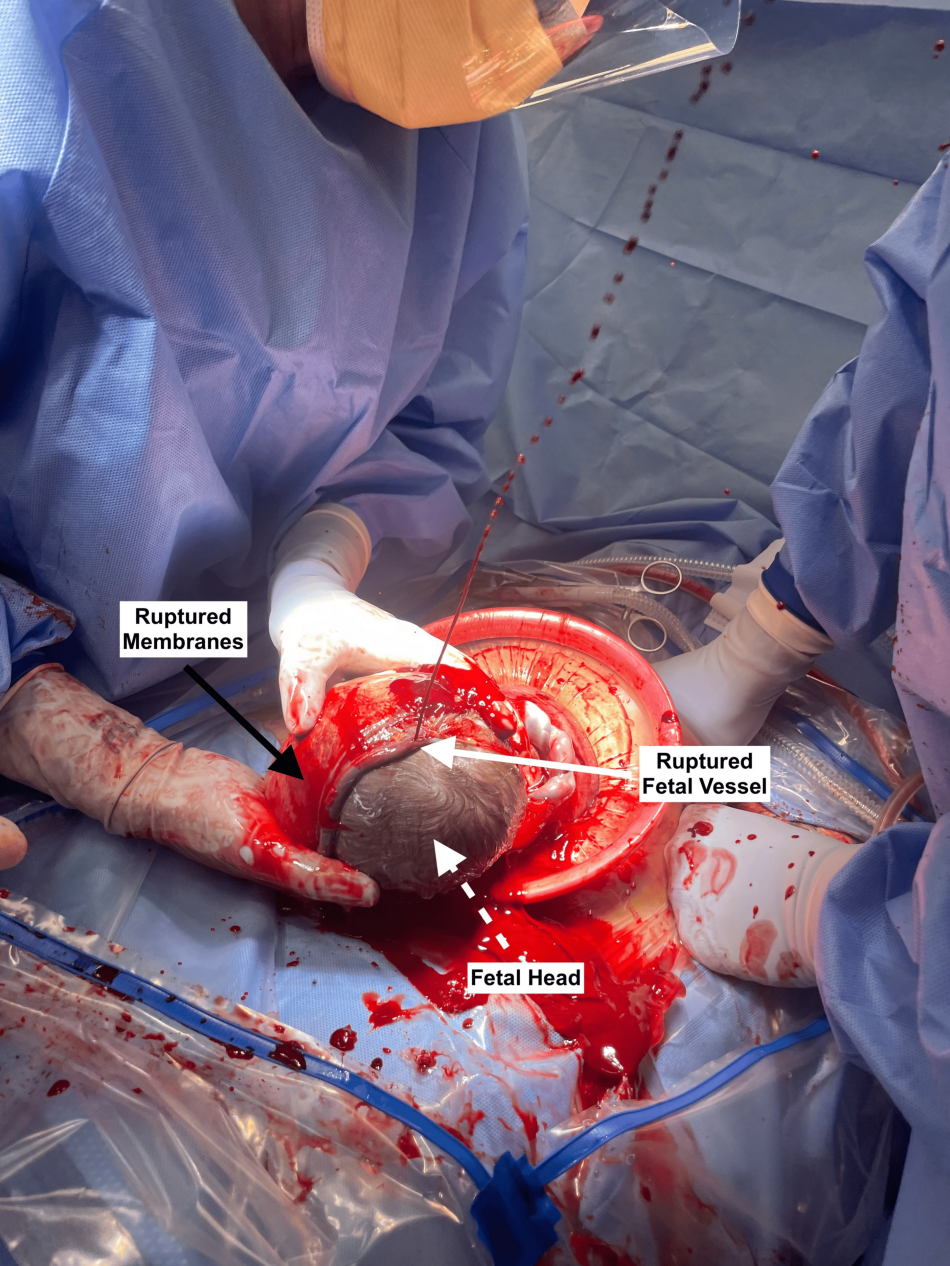
Spontaneous rupture of membranes extending into unprotected fetal vessels of a type I vasa previa during attempted en caul delivery. Spontaneous rupture of membranes (black arrow) during an attempted en caul cesarean delivery for type 1 vasa previa, resulting in rupture of an unprotected fetal vessel (white arrow) overlying the fetal head (white dotted arrow).

The patient remained stable without signs of preterm labor or rupture of membranes. Her cesarean delivery occurred at 35 weeks, as planned. The surgeon carefully incised the uterus, visualizing the unprotected fetal vessels overlying the fetal head (Figure 2). An attempt was made for en caul delivery or delivering the fetus with unruptured membranes. Spontaneous rupture of membranes occurred during the fetal delivery and extended into the unprotected fetal vessels (Figure 3). Rapid and high-pressure bleeding ensued and was managed with expeditious fetal delivery and immediate cord clamping. The neonate was resuscitated in the delivery room and had APGAR scores of 8 and 9 at 1 and 5 minutes, respectively. The neonate was hemodynamically stable and without signs of anemia throughout their admission.

## Discussion

Vasa previa is a diagnosis that can lead to one of the highest-acuity scenarios at a labor and delivery unit, fetal exsanguination. Obstetric trainees are indoctrinated on the urgency of this diagnosis via case scenarios on standardized exams and simulation drills.

This case offers the opportunistic benefit of photographic evidence to demonstrate the brisk fetal bleeding that can occur when a fetal vessel is ruptured. Figure 2 demonstrates the attempted en caul delivery after carefully entering the uterine cavity and exposing the membranes. Figure 3 clearly depicts the high-pressure fetal bleeding after spontaneous rupture of these vessels during an attempt at en caul delivery.

These photographs were validating to the patient as she better understood the reason for her prolonged antepartum admission. She initially was hesitant for prolonged inpatient management, as she was asymptomatic throughout her pregnancy and did not easily perceive the risk surrounding vasa previa. This is not uncommon for obstetric patients faced with the recommendation for a prolonged hospitalization, as these experiences have been associated with anxiety, financial hardship, and social isolation [4,5]. Observational studies by Doyle and Hesson et al. have shown that patient involvement in their care via counseling and feeling listened to can improve their satisfaction [4,5]. We encourage that these photos be used to supplement counseling in a way that engages patients to achieve shared decision making.

With the patient's consent, these photographs were used for multidisciplinary education. These were shared with a team of physicians, nurse midwives, and nurses at a teaching hospital during a daily education meeting. The patient's case was de-identified and presented, using the photographs to illustrate the acuity of vessel rupture.

We consider that these photographs can be incorporated into multidisciplinary obstetric emergency simulations and professional competency exams. In addition, the nursing and medical student clinical rotation experience can be enhanced by reviewing these photographs as part of an obstetric emergency lecture. Future improvements would include recreating this image into a physical model that demonstrates the velocity at which fetal intravascular volume is depleted with vessel rupture during labor or cesarean delivery. 

## Conclusions

This clinical scenario is an excellent demonstration of the continuum of screening, diagnosis, and management of vasa previa. These intraoperative photographs can be used to counsel patients and educate providers and support staff on the potential for sudden acuity that is inherent to vasa previa. We encourage other care teams to consider obtaining intraoperative photographs at the time of a vasa previa cesarean section. 
